# PROFILING THE ORAL MICROBIOME IN OLDER ADULTS WITH DEMENTIA

**DOI:** 10.1093/geroni/igae098.4056

**Published:** 2024-12-31

**Authors:** Sol-ah Jeong, Bock-Young Jung, Insuk Lee, Jun Hyung Cha, Yejun Choi

**Affiliations:** Yonsei University, Seoul, Republic of Korea; Yonsei University College of Dentistry, Seoul, Republic of Korea; Yonsei University, Seoul, Republic of Korea; Yonsei University, Seoul, Republic of Korea; Yonsei University

## Abstract

Early diagnosis and monitoring of clinical progression are crucial for managing dementia. While the relationship between oral health, brain health, and dementia has recently received increased attention, the oral microbiome in dementia patients remains uncharacterized. Therefore, this study aimed to identify the diversity and composition of the oral microbiome in older adults with dementia using next-generation sequencing, and compare it to healthy individuals without dementia to determine whether the oral microbiome could serve as a potential biomarker for the diagnosis and prevention of dementia. A total of 58 older adults aged 65 years and older participated in the study, 30 with dementia and 28 cognitively intact healthy individuals. All participants were screened and clinically diagnosed by a neurologist using the Korean version of the Mini-Mental State Examination (K-MMSE), the Clinical Dementia Rating scale (CDR), and brain magnetic resonance imaging (MRI). 1ml of unstimulated saliva was collected from each subject, the tongue was swabbed with a sterile swab for 20 seconds to collect samples, and full-length 16S rRNA amplicon sequencing was performed on all the samples. Statistical analysis was performed using MaAsLin2, and it was found that Fusobacteriota was more abundant in both the tongue and saliva of the dementia group compared to the healthy group, while Pseudomonadota is more dominant in the healthy group. Fusobacterium periodonticum was found to be more abundant as the K-MMSE scores decreased. Prevotella jejuni, Streptococcus anginosus, and Streptococcus gordonii are more enriched in the Dementia group compared to the healthy group.

